# Supplementary material for: Local and Central Evaluation of HER2 Positivity and Clinical Outcome in Advanced Gastric and Gastroesophageal Cancer—Results from the AGMT GASTRIC-5 Registry
Source: J Clin Med. 2020 Mar 29;9(4):935. doi: 10.3390/jcm9040935 (PMC7230156; doi:10.3390/jcm9040935)
Supplement: Supplementary file 1 [file jcm-09-00935-s001.pdf]

Supplementary files

Supplementary Table 1. Applied HER2 test kits by local pathology laboratories and respective time frames.

**Methods Local Pathology**	**Number of Local Pathologies, Year of Local Testing (Timeframe)**
HER2 status by immunohistochemistry (IHC)	
HER-2/neu (4B5) Rabbit Monoclonal Primary Antibody, PATHWAY; Roche Ventana	7 sites, 2006–2018
HerceptTest™; DAKO	2 sites, 2010–2018
LDT c-erbB-2 Oncoprotein (HER-2), clone CB11 stained on DAKO Autostainer Plus	1 site, 2010–2012
In situ hybridization (ISH), in equivocal cases (IHC score 2+)	
Ventana Inform HER-2 Dual ISH DNA Probe Cocktail Assay (Roche)	3 sites, 2011–2018
PathVysion HER-2 DNA Probe Kit (Abbott)	3 sites, 2012–2017
HER2 IQFISH pharmDx - Dako Agilent	1 site, 2011

Supplementary Table 2. Comparison HER2 immunohistochemistry results between local and central laboratories.

n = 183		**Local HER2 IHC (%)**			
		**0**	**1+**	**2+**	**3+**
Central HER2 IHC	**0** (*n* = 43)	35 (81)	5 (12)	2 (5)	1 (2)
	**1+** (*n* = 91)	51 (56)	19 (21)	17 (19)	4 (4)
	**2+** (*n* = 13)	5 (38)	0 (0)	3 (24)	5 (38)
	**3+** (*n* = 36)	1 (3)	1 (3)	4 (11)	30 (83)

IHC: immunohistochemistry.

Supplementary Table 3. Comparison of local and central HER2 ISH result.

	**Central HER2 ISH**	***n* = 41 (%)**
Positive	Negative	3 (7)
Positive	Not performed	6 (15)
Negative	Not performed	15 (37)
Not performed	Positive	1 (2)
Not performed	Negative	13 (32)
Positive	Positive	1 (2)
Negative	Negative	2 (5)

ISH: in-situ hybridization.
